# MiR-21-mediated Metabolic Alteration of Cancer-associated Fibroblasts and Its Effect on Pancreatic Cancer Cell Behavior: Retraction

**DOI:** 10.7150/ijbs.91613

**Published:** 2024-02-09

**Authors:** Shuo Chen, Xi Chen, Tao Shan, Jiancang Ma, Wanrun Lin, Wei Li, Ya'an Kang

**Affiliations:** 1 Department of General Surgery, The Second Affiliated Hospital of Medical College, Xi'an Jiaotong University, Xi'an, Shaanxi 710004 China;; 2 Department of Pathology, Fudan University Shanghai Cancer Center, Shanghai 200032 China;; 3 Graduate School, Fourth Military Medical University, Xi'an 710033, China.; 4 Department of Surgical Oncology, The University of Texas MD Anderson Cancer Center, Houston, Texas, United States of America.

The authors noticed the presence of some unreliable data during the experimental validation process and have made the decision to retract the paper. We deeply regret any inconvenience stemming from our oversight and sincerely apologize for any unintended errors. All authors are in unanimous agreement regarding the retraction of the article.

